# Regulation of RhoA activity by the cellular prion protein

**DOI:** 10.1038/cddis.2017.37

**Published:** 2017-03-16

**Authors:** Hee-Jun Kim, Hong-Seok Choi, Jeong-Ho Park, Mo-Jong Kim, Hyoung-gon Lee, Robert Bob Petersen, Yong-Sun Kim, Jae-Bong Park, Eun-Kyoung Choi

**Affiliations:** 1 Ilsong Institute of Life Science, Hallym University, Anyang, Republic of Korea; 2 Department of Microbiology, College of Medicine, Hallym University, Chuncheon, Republic of Korea; 3 Department of Biomedical Gerontology, Graduate School of Hallym University, Chuncheon, Republic of Korea; 4 Department of Biology, The University of Texas at San Antonio, San Antonio, TX, USA; 5 Department of Pathology, Case Western Reserve University, Cleveland, OH, USA; 6 Departments of Neuroscience and Neurology, Case Western Reserve University, Cleveland, OH, USA; 7 Department of Biochemistry, College of Medicine, Hallym University, Chuncheon, Republic of Korea

## Abstract

The cellular prion protein (PrP^C^) is a highly conserved glycosylphosphatidylinositol (GPI)-anchored membrane protein that is involved in the signal transduction during the initial phase of neurite outgrowth. The Ras homolog gene family member A (RhoA) is a small GTPase that is known to have an essential role in regulating the development, differentiation, survival, and death of neurons in the central nervous system. Although recent studies have shown the dysregulation of RhoA in a variety of neurodegenerative diseases, the role of RhoA in prion pathogenesis remains unclear. Here, we investigated the regulation of RhoA-mediated signaling by PrP^C^ using both *in vitro* and *in vivo* models and found that overexpression of PrP^C^ significantly induced RhoA inactivation and RhoA phosphorylation in hippocampal neuronal cells and in the brains of transgenic mice. Using siRNA-mediated depletion of endogenous PrP^C^ and overexpression of disease-associated mutants of PrP^C^, we confirmed that PrP^C^ induced RhoA inactivation, which accompanied RhoA phosphorylation but reduced the phosphorylation levels of LIM kinase (LIMK), leading to cofilin activation. In addition, PrP^C^ colocalized with RhoA, and the overexpression of PrP^C^ significantly increased neurite outgrowth in nerve growth factor-treated PC12 cells through RhoA inactivation. However, the disease-associated mutants of PrP^C^ decreased neurite outgrowth compared with wild-type PrP^C^. Moreover, inhibition of Rho-associated kinase (ROCK) substantially facilitated neurite outgrowth in NGF-treated PC12 cells, similar to the effect induced by PrP^C^. Interestingly, we found that the induction of RhoA inactivation occurred through the interaction of PrP^C^ with RhoA and that PrP^C^ enhanced the interaction between RhoA and p190RhoGAP (a GTPase-activating protein). These findings suggest that the interactions of PrP^C^ with RhoA and p190RhoGAP contribute to neurite outgrowth by controlling RhoA inactivation and RhoA-mediated signaling and that disease-associated mutations of PrP^C^ impair RhoA inactivation, which in turn leads to prion-related neurodegeneration.

The activity of Rho GTPases (Rho, Rac, and Cdc42) is controlled by regulatory proteins that cycle between an inactive GDP-bound state and an active GTP-bound state. Rho GTPases are activated by guanine nucleotide exchange factors (GEFs), which catalyze the exchange of GDP for GTP. In contrast, GTPase-activating proteins (GAPs), which stimulate Rho GTPase activity, and Rho guanine nucleotide dissociation inhibitors (GDIs), which inhibit the exchange of GDP for GTP in the cytoplasm by forming a Rho–RhoGDI complex, induce inactivation state of these GTPases.^1, 2^ Furthermore, the Rho–RhoGDI complex needs to be dissociated by GDI displacement factor (GDF) before Rho GTPases are activated by GEFs.^3^ Activated Rho GTPases stimulate effector proteins, such as Rho-associated kinase (ROCK), mDia, and p21-activated kinase (PAK). Rho GTPases have roles in a variety of cellular functions including cytoskeletal rearrangement.^4^ In particular, the Ras homolog gene family member A (RhoA) and RhoA regulatory proteins (including p190RhoGAP and RhoGDI) participate in neuronal differentiation processes, such as neurite outgrowth, neuronal migration, axonal growth, and dendritic spine formation and maintenance.^5^ In addition, several studies have shown that RhoA inactivation is essential for neuronal morphogenesis.^6, 7^ Application of C3 toxin (a RhoA inhibitor) or Y27632 (a ROCK inhibitor) and overexpression of dominant-negative mutant RhoA enhanced neurite outgrowth from PC12 cells in response to nerve growth factor (NGF), basic fibroblast growth factor (bFGF), and cAMP.^8, 9^

The cellular prion protein (PrP^C^) is a cell-surface glycosylphosphatidylinositol (GPI)-anchored glycoprotein attached to the plasma membrane.^10^ PrP^C^ has been associated with various cellular functions, including the cell cycle, cell growth, cell proliferation, cell–cell adhesion, cell migration, and the maintenance of cell shape.^11, 12^ PrP^C^ is strongly expressed in the central nervous system (CNS) and can act as a regulator of neuronal development, differentiation, and neurite outgrowth, which may depend on interactions with various regulatory proteins, including heparan sulfate proteoglycans,^13, 14^ stress-inducible protein-1,^15^ Grb2 protein,^16^ caveolin,^17^ neural cell adhesion molecules (NCAMs),^18, 19^ and extracellular matrix (ECM) proteins.^20, 21^ In addition, PrP^C^ exerts its functions by interacting with several kinases, including Fyn, protein kinase C (PKC), protein kinase A (PKA), phosphatidylinositol-3-kinase (PI3K)/Akt, and extracellular regulated kinases (ERK1/2).^22, 23^

Loss of PrP^C^ function has been implicated in neuronal polarization and neurite outgrowth through the modulation of integrin–ECM interactions and the RhoA-ROCK-LIM kinase (LIMK)-cofilin signaling pathway.^24^ Recently, ROCK overactivation and ROCK-3-phosphoinositide-dependent kinase 1 (PDK1) complex formation were shown to contribute to the regulation of neuronal polarity and the generation of pathogenic prions.^25^ However, the functional interaction between PrP and RhoA-related signaling molecules remains unknown.

In this study, we investigated the relationships of PrP^C^ expression with RhoA activity and neurite outgrowth. We demonstrated that PrP^C^ induced neurite outgrowth by inactivating RhoA and that PrP^C^-mediated RhoA inactivation may be achieved by the interaction of PrP with RhoA and/or p190RhoGAP, resulting in the phosphorylation of RhoA at Ser188.

## Results

### PrP^C^ regulates RhoA activation and RhoA-mediated signaling

**Figure 1:**
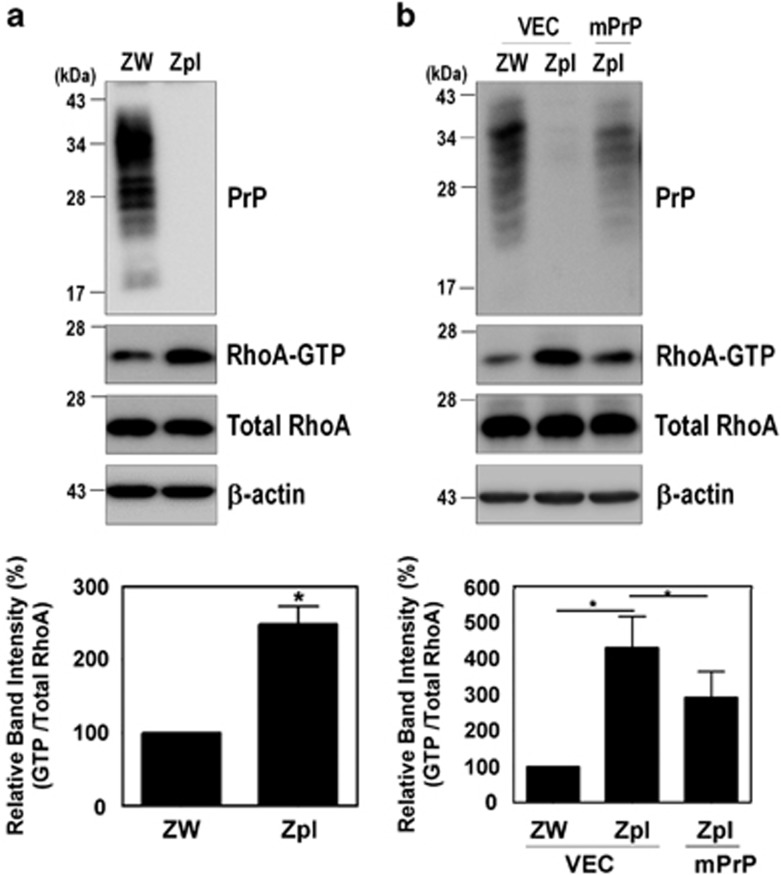
PrP^C^ regulates RhoA activation. (**a** and **b**) Detection of RhoA-GTP by GST-Rhotekin-RBD pull-down assay in cells expressing PrP^C^ (ZW) and PrP knockout (Zpl) with or without expressing mPrP. The level of RhoA-GTP was determined by western blot with anti-RhoA antibody following a pull-down assay. The data are expressed as the mean±S.E. of three independent experiments (*P<0.05, *n*=3)

To determine whether the PrP^C^ affects RhoA activity, a pull-down assay was performed with the glutathione-*S*-transferase (GST)-Rhotekin-Rho-binding domain (RBD) in the ZW13-2 (wild-type, WT) and Zpl3-4 (PrP knockout) mouse hippocampal neuronal cell lines (Supplementary Figure 1), as previously established.^26^ We found that the level of RhoA-GTP in PrP knockout Zpl cells was significantly higher than in control ZW cells (Figure 1a). We confirmed this result by re-introducing mouse PrP (mPrP) into Zpl cells, which exhibited lower RhoA-GTP levels than Zpl cells that expressed the empty vector alone (Figure 1b). These results suggest that PrP^C^ negatively regulates RhoA activity in hippocampal neuronal cells.

**Figure 2:**
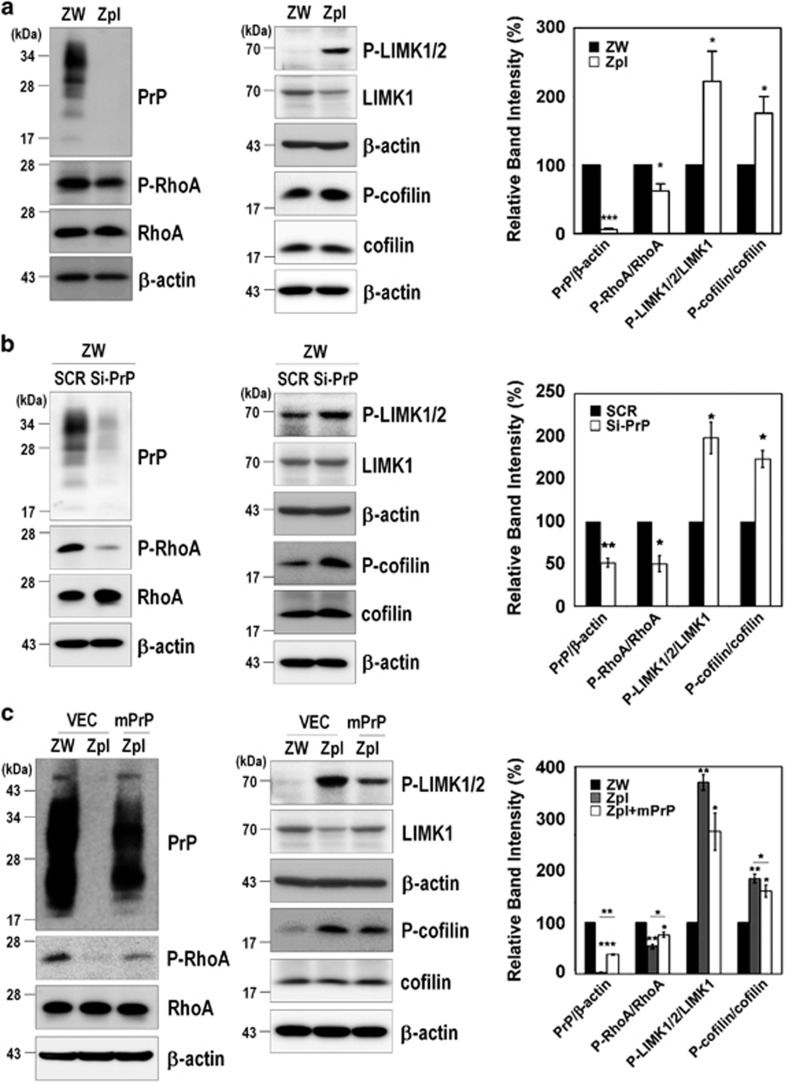
PrP^C^ modulates the RhoA-ROCK-LIMK-cofilin pathway. (**a-c**) Phosphorylation of RhoA, LIMK1/2, and cofilin in ZW and Zpl cells (**a**), in ZW cells transfected with scrambled RNA (SCR) or mPrP-targeted siRNA (Si-PrP) (**b**) and in Zpl cells with or without expressing mPrP (**c**) was analyzed in triplicate by western blot. The intensities of the bands in each panel were measured and quantified for each group, and the values are expressed as the mean±S.E. of three independent experiments (*P<0.05, **P<0.01, ***P<0.001, *n*=3)

To further investigate the signaling pathway of RhoA regulated by PrP^C^ expression, we determined whether PrP^C^ modulates the RhoA-ROCK-LIMK-cofilin pathway. As shown in Figure 2, PrP knockout and siRNA-mediated knockdown of endogenous mPrP (si-mPrP^C^) cells exhibited less phosphorylated RhoA at Ser188 (p-RhoA), which negatively regulates RhoA activity by enhancing its interaction with RhoGDI and translocates RhoA from the membrane to the cytosol^27^ with increases in phospho-LIMK1/2 (p-LIMK1/2) and phospho-cofilin (p-cofilin) (Figures 2a and b). Supporting these results, the re-introduction of mPrP reversed the changes in the levels of p-RhoA, p-LIMK1/2, and p-cofilin compared with Zpl cells expressing the empty vector alone, yielding a result similar to that observed for the ZW cells (Figure 2c).

**Figure 3:**
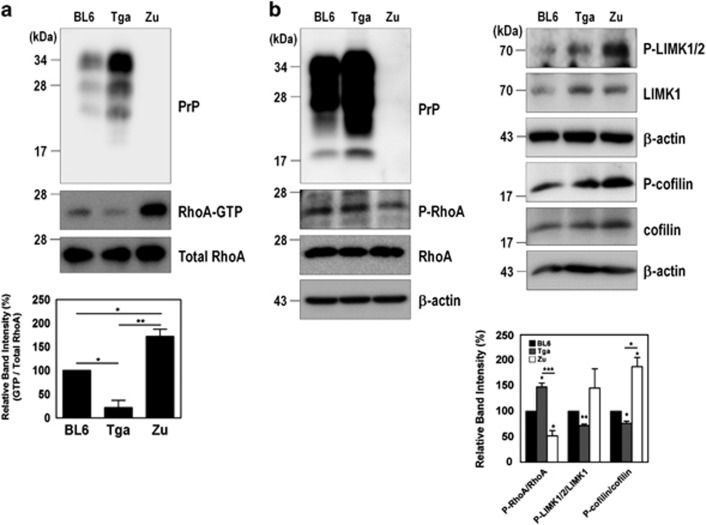
PrP^C^ is involved in RhoA inactivation in the brains of three different types of mice. (**a**) Detection of RhoA-GTP levels in the brains of C57BL/6J (BL6, WT), Tga20 (Tga, PrP overexpression) and Zürich I (Zu, PrP-deficient) mice (*n*=3 per each group). (**b**) Phosphorylation of RhoA, LIMK, and cofilin was assessed in the whole-brain lysates of C57BL/6J, Tga20, and Zürich I mice. The data are expressed as the mean±S.E. of three independent experiments (*P<0.05, **P<0.01, ***P<0.001, *n*=3)

To confirm these results, we examined the effect of PrP^C^ expression on RhoA activity and on the phosphorylation levels of RhoA downstream proteins in the brains of three different types of mice: WT (C57BL/6J) mice, Tga20 mice that overexpress PrP^C^ (Tga20), and Zürich I *Prnp*-deficient (Zürich I) mice that lack PrP^C^. As expected, we observed an increase in RhoA-GTP level (Figure 3a) accompanied by a decrease in p-RhoA and increases in both p-LIMK1/2 and p-cofilin (Figure 3b) in the brains of the Zürich I mice compared with the brains of the WT and Tga20 mice. These findings suggest that the expression of PrP^C^ inactivates RhoA activity and subsequently affects its downstream regulatory proteins including LIMK and cofilin.

### PrP^C^ controls F-actin formation through the RhoA/ROCK pathway

**Figure 4:**
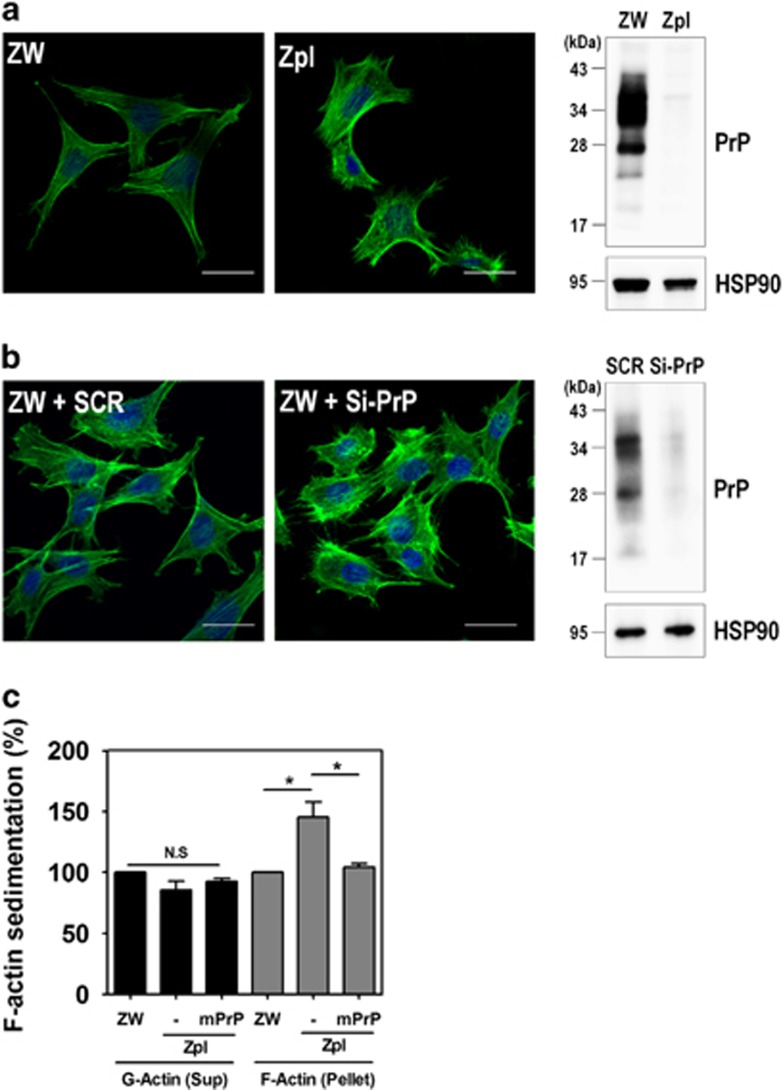
Depletion of PrP^C^ increases F-actin formation. (**a** and **b**) Immunocytochemical staining for F-actin in ZW and Zpl cells (**a**), and ZW cells transfected with either scrambled RNA (SCR) or mPrP-targeted siRNA (Si-PrP) (**b**) using Alexa Fluor 488-phalloidin (green). DAPI (blue) was used to counterstain the nuclei. All pictures are representative of multiple images from three independent experiments (scale bars, 20 *μ*m). The expression of PrP^C^ was determined by western blot with anti-PrP (3F10) antibody and HSP90 was used as a loading control. (**c**) The expression of F-actin assessed by a sedimentation assay in ZW, Zpl, and Zpl expressing mPrP cells was analyzed by western blot with anti-*β*-actin, anti-PrP (3F10) and anti-HSP90 antibodies. The intensities of the bands in each panel were measured and quantified for each group, and the values are expressed as the mean±S.E. of three independent experiments (*P<0.05, *n*=3)

Previous studies have reported that RhoA activation has a role in the regulation of cytoskeleton reorganization through the formation of actin stress fibers and focal adhesions.^28, 29^ Thus, we investigated the effect of PrP^C^ on the formation of actin stress fibers in ZW and Zpl cells. Stress fibers were observed to form filamentous actin (F-actin), which was detected with fluorescein isothiocyanate (FITC)-conjugated phalloidin. As shown in Figure 4a, F-actin formation was more strongly detected in Zpl cells than in ZW cells, and silencing PrP^**C**^ in ZW cells markedly enhanced F-actin formation (Figure 4b). To confirm this finding, we determined the changes in G-actin and F-actin levels in ZW, Zpl, and Zpl cells expressing mPrP using G-actin/F-actin sedimentation assay. Consistent with the results of F-actin formation, PrP knockout (Zpl cells) resulted in significantly increased F-actin sedimentation in the pellet fraction, whereas G-actin levels were not changed in the supernatant fraction (Figure 4c). To further elucidate whether F-actin formation regulated by PrP^C^ is due to RhoA-mediated signaling, cells were treated with Y27632, an inhibitor of ROCK. Interestingly, Y27632 treatment decreased F-actin formation in ZW cells (Supplementary Figure 2a). In addition, we analyzed PrP^C^ on F-actin-mediated cell adhesion using WST-1 reagent, which is a quantitative method for evaluating attached cells. In a cell adhesion assay, F-actin-mediated cell adhesion was significantly decreased in Zpl cells than ZW or Zpl cells expressing mPrP (Supplementary Figure 3). These findings indicate that PrP^C^ is involved in F-actin formation and cell adhesion through the RhoA/ROCK signaling pathway.

### PrP^C^ interacts with both RhoA and p190RhoGAP

**Figure 5:**
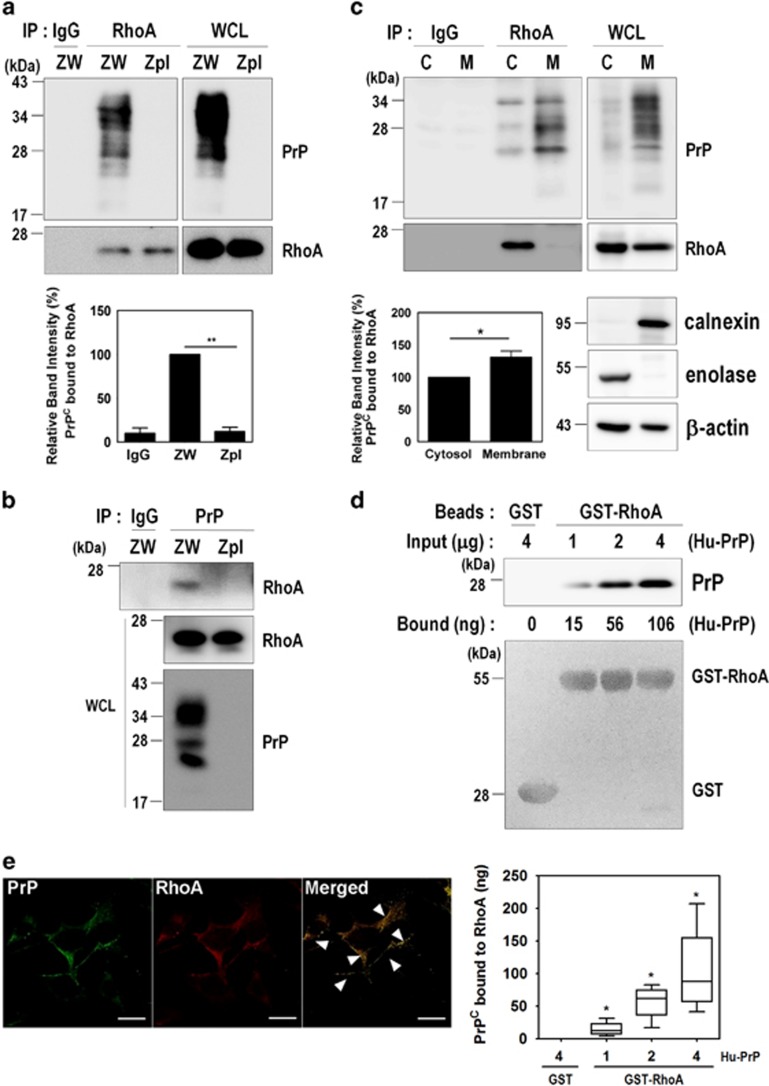
PrP^C^ interacts with RhoA. (**a **and** b**) Co-immunoprecipitation of PrP with RhoA using ZW and Zpl cell lysates were performed with either anti-RhoA (**a**) or anti-PrP (3F10) (**b**) antibodies, and then analyzed by western blot with anti-PrP and anti-RhoA antibodies, respectively. WCL, whole-cell lysates. (**c**) The subcellular fractions from ZW cells were used to immunoprecipitate RhoA with anti-RhoA antibody and then analyzed by western blot with anti-PrP (3F10) and anti-RhoA antibodies. Enolase and calnexin were used as makers for the cytosol (C) and membrane (M) fractions, respectively. *β*-Actin as a loading control. (**d**) GST and GST-RhoA beads were incubated with human recombinant PrP (Hu-PrP) as indicated, and the level of Hu-PrP bound to GST-RhoA was determined by western blot with anti-PrP (3F4) antibody. The boxplot showing the means±S.E. of abundance of the PrP-RhoA complex, was calculated from the BSA standard curve in three (*n*=3) independent experiments. The GST and GST-RhoA samples were stained with Ponceau S to confirm the equal loading. (**e**) Colocalization of PrP with RhoA was assessed by double immunofluorescence staining and confocal microscopy. All above data are expressed as the mean±S.E. of three independent experiments (*P<0.05, **P<0.01, *n*=3)

To identify the molecular mechanism by which PrP^**C**^ induces RhoA inactivation, we sought to determine whether PrP^**C**^ and RhoA directly interact in ZW and Zpl cells. As PrP^**C**^ possesses a partial sequence homology with RhoA and RhoA effector proteins, including rhotekin, ROCK1, protein kinase N (PKN), and rhophilin (Supplementary Figure 5), we confirmed the interaction of PrP^C^ with RhoA using a co-immunoprecipitation assay in ZW cells (Figures 5a and b). To further verify whether the interaction between PrP^C^ and RhoA occurs in the cytosol or membrane fractions in ZW cells, co-immunoprecipitation of RhoA and PrP^C^ was conducted on both fractions. As shown in Figure 5c, the interaction between PrP^C^ and RhoA in the membrane fraction was slightly increased compared with the cytosol fraction, although the level of RhoA in the cytosol fraction was higher than the level in the membrane fraction (Figure 5b). Furthermore, purified human recombinant PrP^C^ protein directly bound to purified recombinant GST-RhoA protein in a concentration-dependent manner (Figure 5d). We also found that PrP^C^ was colocalized with RhoA in the cytoplasm and the plasma membrane of ZW cells (Figure 5e, arrowheads), suggesting that PrP^C^ directly interacts with RhoA in both the cytoplasm and the membrane.

**Figure 6:**
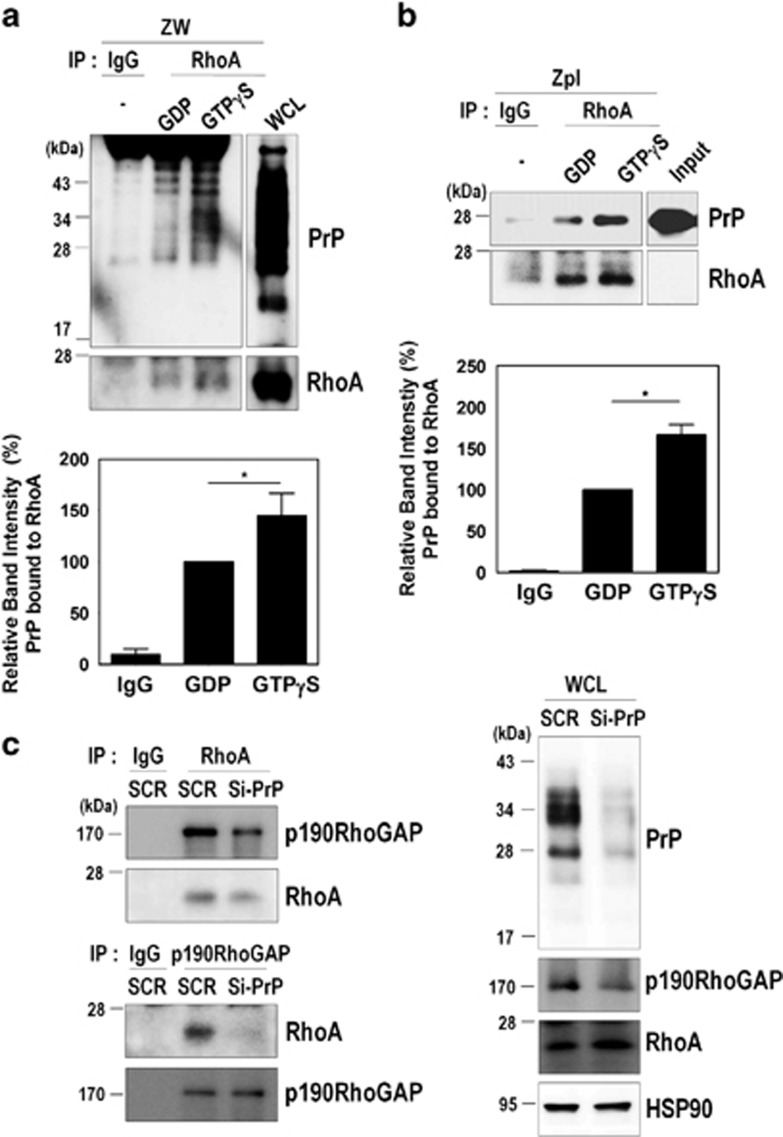
PrP^C^ binds to GTP-bound RhoA and p190RhoGAP. (**a**) ZW cell lysates were preloaded with GDP or GTP*γ*S followed by immunoprecipitation with the anti-RhoA antibody and analyzed by western blot using the anti-PrP (3F10) and anti-RhoA antibodies. WCL, whole-cell lysates. (**b**) Zpl cell lysates preloaded with GDP or GTP*γ*S were immunoprecipitated with anti-RhoA antibody, incubated with 2 *μ*g of human recombinant PrP (Hu-PrP), and analyzed by western blot with the anti-PrP (3F4) and anti-RhoA antibodies. (**c**) The co-immunoprecipitation of RhoA or p190RhoGAP using ZW cells transiently transfected with either SCR or Si-PrP was detected by western blot using anti-p190RhoGAP and anti-RhoA antibodies, respectively. The data are expressed as the mean±S.E. of three independent experiments (*P<0.05, *n*=3)

As RhoA functions as a molecular switch between active GTP-bound and inactive GDP-bound states, we next investigated whether the GDP- or GTP-bound states of RhoA affect its interaction with PrP^C^. ZW cell lysates were preloaded with either GDP or GTP*γ*S, and then co-immunoprecipitation of RhoA with PrP was performed. We found that PrP^C^ preferentially interacts with active GTP*γ*S-bound RhoA compared with GDP-bound RhoA in ZW cells (Figure 6a). In addition, the interaction of purified human recombinant PrP^C^ with RhoA was also increased in the presence of GTP*γ*S in ZPL cells (Figure 6b). These results showed that PrP^C^ induced RhoA inactivation through a direct interaction with RhoA in the cytosol and membrane fractions of PrP^C^-expressing cells and that GTP-bound RhoA may more favorably interact with PrP^C^.

The p190RhoGAP is known to be a major regulator of RhoA activity,^30, 31^ it contributes to actin rearrangement and neurite outgrowth through binding to GTP-bound RhoA and subsequently enhancing the hydrolysis of GTP.^32^ Thus, we examined whether PrP^C^ regulates RhoA inactivation by facilitating the interaction between RhoA and p190RhoGAP. As expected, reducing PrP^C^ expression by si-PrP^C^ decreased its interactions with both RhoA and p190RhoGAP (Figure 6c). These findings indicate that PrP^**C**^ interacts with RhoA, as well as p190RhoGAP, and that PrP^C^ mediates the interaction between RhoA and p190RhoGAP.

### The disease-associated PrP^C^ mutants impair neurite outgrowth

**Figure 7:**
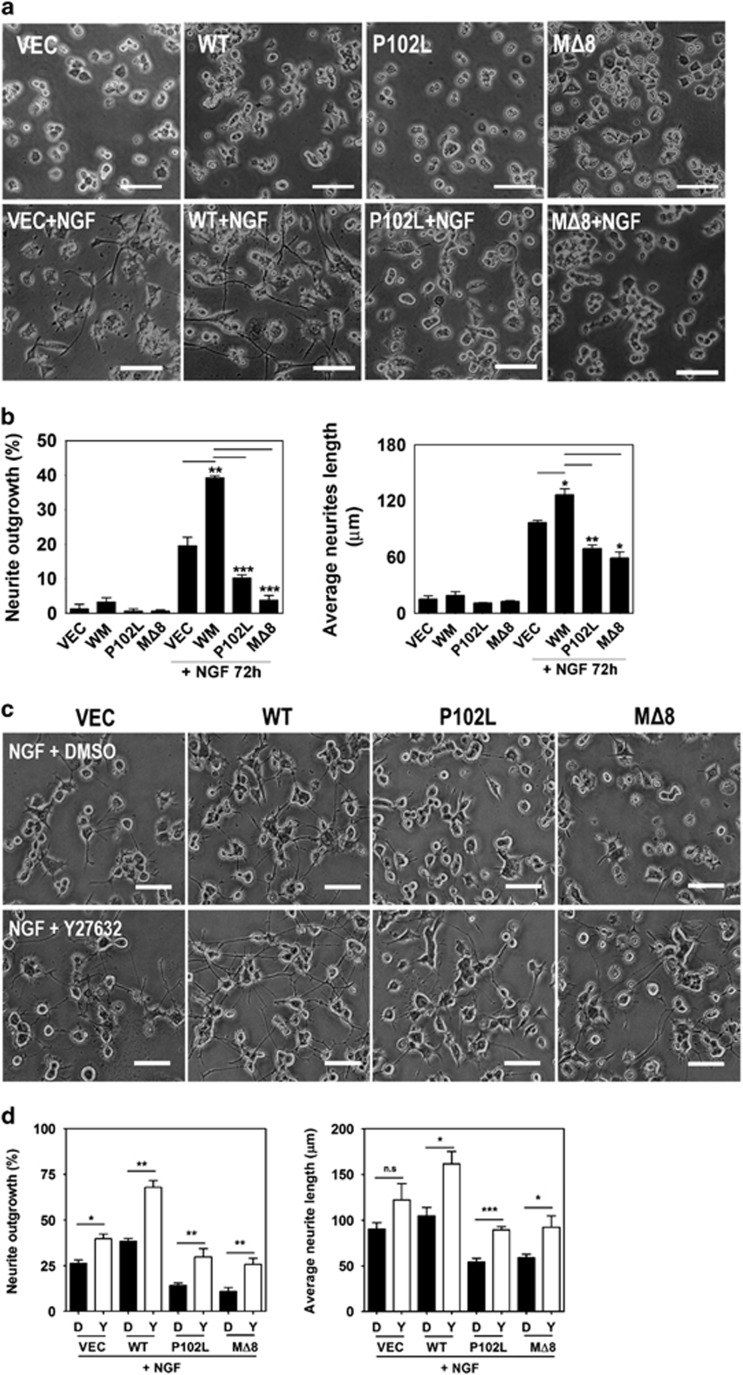
The disease-associated mutations of PrP^C^ impair neurite outgrowth. (**a** and **b**) PC12 cells stably expressing either vector, WT, P102L, or MΔ8 were treated with 50 ng/ml NGF for 72 h. (**c** and **d**) The cells expressing either vector, WT, P102L, or MΔ8 were incubated with or without 10 *μ*M Y27632 in the presence of NGF. Changes in the cell morphology, neurite length, and neurite numbers were determined under a microscope. The data are expressed as the mean±S.E. of three independent experiments (*P<0.05, **P<0.01, ***P<0.001, *n*=3)

Point mutations and polymorphisms of PrP^C^ are associated with genetic prion diseases,^33^ and several studies have shown an association between the pathogenicity of prion diseases and neuronal differentiation.^34, 35^ Therefore, we investigated whether the disease-associated mutations of PrP^C^ affect NGF-induced neurite outgrowth in PC12 cells stably expressing WT or disease-associated mutants of PrP^C^ (P102L and MΔ8). Interestingly, the PC12 cells expressing WT PrP^C^ exhibited enhanced neurite outgrowth and neurite length in response to NGF, whereas the cells expressing disease-associated PrP^C^ mutants impaired neurite outgrowth and reduced neurite length (Figures 7a and b). In addition, the inhibition of ROCK by Y27632 treatment significantly enhanced neurite outgrowth and neurite length (Figures 7c and d). Using mutants of RhoA (S188D, mimicking the phosphorylated form; S188A, mimicking the dephosphorylated form), we found that RhoA phosphorylation (Ser188) induced neurite outgrowth in NGF-differentiated PC12 cells expressing PrP^C^ (Supplementary Figure 4). These data indicate that PrP^C^ may facilitate neurite outgrowth and affect the cellular signal transduction related to RhoA inactivation and that the phosphorylation of RhoA at Ser188 can also enhance PrP^C^-mediated neurite outgrowth.

### The disease-associated mutations of PrP^C^ affect RhoA signaling through reduced interaction with RhoA and p190RhoGAP

**Figure 8:**
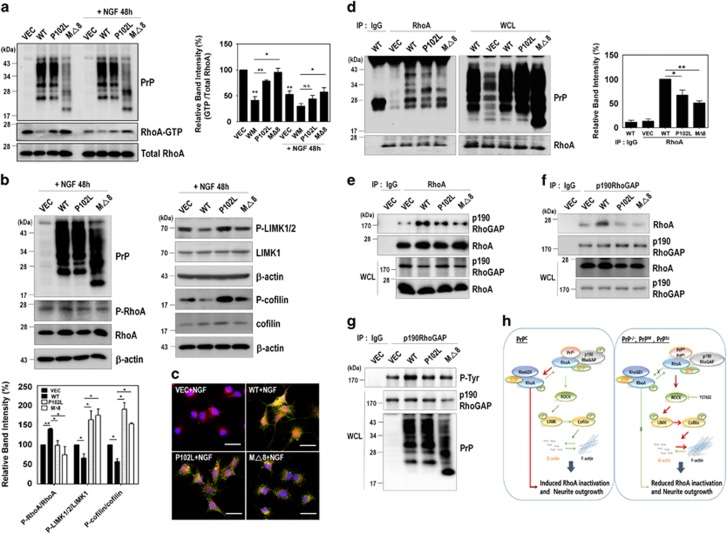
The disease-associated mutants of PrP^C^ affect RhoA signaling through the reduced interactions with RhoA and p190RhoGAP. (**a** and **b**) The level of RhoA-GTP following a pull-down assay (**a**) and the phosphorylation of RhoA, LIMK, and cofilin (**b**) was analyzed by western blot in PC12 cells expressing either vector, WT, P102L, or MΔ8 in response to NGF. (**c**) Colocalization of PrP with RhoA in the NGF-treated PC12 cells expressing either vector, WT, P102L, or MΔ8 was determined using confocal microscopy (green, PrP; red, RhoA; blue, DAPI). (**d**) HEK293 cells were transiently transfected with either vector, WT, P102L, or MΔ8. The co-immunoprecipitation of RhoA was detected by western blot using anti-PrP (3F4) and anti-RhoA antibodies. (**e**-**g**) PC12 cells transiently transfected WT or disease-associated mutants of PrP^C^ were lysed and immunoprecipitated with anti-RhoA (**e**) and p190RhoGAP antibodies (**f**). (**g**) The p190RhoGAP phosphorylation (p-Tyr) was detected using p-Tyr antibody after p190RhoGAP immunoprecipitation. All above data are expressed as the mean±S.E. of three independent experiments (*P<0.05, **P<0.01, *n*=3). (**h**) PrP^C^–RhoA interaction stimulates RhoA inactivation and neurite outgrowth. In PrP^C^-expressing cells and mice, PrP^C^ increased the phosphorylation of RhoA and p190RhoGAP, enhancing the interaction between RhoA and p190RhoGAP. This complex led to the inactivation of RhoA and its downstream effectors. Subsequently, RhoA inactivation decreased actin polymerization and enhanced neurite outgrowth. In contrast, depleting PrP^C^ or expressing disease-associated mutants of PrP^C^ prevented RhoA inactivation and neurite outgrowth by interfering with the interaction

To investigate the effect of disease-associated mutations of PrP^C^ on RhoA activity, PC12 cells were transiently transfected with an empty vector, WT PrP^C^, or disease-associated mutants of PrP^C^, and then treated with NGF. Interestingly, we observed that RhoA-GTP levels were increased in PC12 cells expressing disease-associated mutants of PrP^C^ compared with the cells expressing WT PrP^C^, although these changes were lower in the presence of NGF (Figure 8a). Interestingly, decrease in p-RhoA and increases in both p-LIMK1/2 and p-cofilin were detected in the cells expressing disease-associated mutants of PrP^C^ compared with the cells expressing WT PrP^C^ (Figure 8b). These results, which are correlated with those in PrP knockout or knockdown cells, indicate that PrP^C^ regulates neurite outgrowth through inactivation of RhoA and the Rho/ROCK signaling pathway. Next, we examined whether these disease-associated mutations of PrP^C^ affect the interactions between not only PrP^C^ and RhoA but also RhoA and p190RhoGAP. We found that the colocalization of PrP with RhoA was significantly decreased in the cells expressing disease-associated mutants of PrP^C^ compared with cells expressing PrP^C^ WT based on immunofluorescence staining (Figure 8c). Consistently, the co-immunoprecipitation of RhoA with the disease-associated mutants of PrP^C^ was significantly decreased (Figure 8d). Moreover, the overexpression of disease-associated PrP^C^ mutants markedly decreased its interaction with RhoA and p190RhoGAP (Figures 8e and f). Notably, the disease-associated mutations of PrP^C^ reduced p190RhoGAP tyrosine phosphorylation, which led to a decrease in p190RhoGAP activity (Figure 8g). Taken together, these findings suggest that the disease-associated mutations of PrP^C^ impaired RhoA signaling and the interaction with RhoA and p190RhoGAP.

## Discussion

The physiological activity of PrP^C^ in many important aspects of cell biology, including neuritogenesis and cell signaling, has been well established.^24, 25, 36^ Recent studies have demonstrated that PrP^C^ contributes to neuritogenesis through modulating the *β*1 integrin-coupled RhoA-ROCK-LIMK-cofilin signaling axis^24^ and that prion-induced ROCK overactivation is involved in neuronal polarity and prion pathogenesis.^25^ However, it is still unclear whether PrP^C^ can directly regulate RhoA activity, and its related effector proteins have not yet been elucidated.

In this study, we discovered a novel mechanism by which PrP^C^ controls RhoA activity and the RhoA-mediated signaling pathway (Figure 8h). Both knockdown and silencing of PrP^C^ induce activation of RhoA, which is best known for its function in reorganizing the actin cytoskeleton into stress fibers and focal adhesions,^5^ in concert with altered activities of downstream effector proteins (i.e., LIMK and cofilin). In addition, PrP^C^ expression is also involved in the regulation of focal adhesion dynamics and actin polymerization.^37, 38^ We also found that the depletion of PrP^C^ or the expression of disease-associated PrP^C^ mutants impaired actin cytoskeleton dynamics and inhibited neurite outgrowth, possibly via increased phosphorylation of cofilin (an inactive form), leading to microfilaments that support stabilization. Unphosphorylated cofilin (an active form) is known to sever F-actin, resulting in depolymerization of F-actin.^29^

The altered balance of cofilin activity is critical for the regulation of actin cytoskeleton dynamics and has been associated with neurodegeneration.^39, 40^ NADPH oxidase (NOX) is activated through a PrP^C^-dependent pathway in response to proinflammatory cytokines,^41^ and the overexpression of PrP^C^ alone also induced NOX-mediated ROS generation leading to the activation of cofilin and its oxidation (Cys39 and Cys147) followed by cofilin-actin rod formation.^42^ We found that overexpression of PrP^C^ significantly reduced the amount of p-cofilin, leading to cofilin activation without changes in the total level of cofilin through the RhoA-ROCK-LIMK-cofilin pathway, and led to increased neurite outgrowth in NGF-treated PC12 cells. In addition, this regulation depends on the membrane environment and the interactions among membrane components (i.e., NOX isoforms, *β*1 integrin, laminin, and fyn), resulting in PrP^C^-dependent neuronal differentiation or synaptic dysfunction.

PrP^C^ has been implicated in neurite outgrowth as an interacting partner with NCAM and laminin.^18, 19, 43^ In addition, several interacting partners have been reported to directly bind to PrP^C^, which enhances brain development, neuronal differentiation, and neuronal cell death in various cell lines and animal models.^13, 14, 15, 16, 17, 18, 19, 20, 21^ Moreover, these interactions can regulate various signaling pathways, such as PI3K/AKT,^22, 44^ ERK1/2,^22, 23^ and RhoA/Rac1/Cdc42.^12^ Interestingly, the PI3K/Akt and ERK1/2 pathways regulate transcriptional profiles that promote neurite extension.^45^ Activation of Rac1 and Cdc42 in conjunction with inhibition of RhoA activity increases neurite extension via posttranslational mechanisms – both pathways functionally connect with ROCK.^46^ We also demonstrated increased neurite extension and neurite length as a result of ROCK inhibition by Y27632, suggesting that PrP^C^ exerts its influence on neuronal differentiation by modulating RhoA-mediated signaling effectors (i.e., ROCK and p190RhoGAP).

Specifically, we demonstrated the biological consequences of PrP^C^-mediated RhoA inactivation that results from the interaction of PrP^C^ with RhoA and p190RhoGAP, and overexpressing PrP^C^ results in increased tyrosine phosphorylation of p190RhoGAP, which elevates p190RhoGAP activity. Indeed, p190RhoGAP was reported to be activated through tyrosine phosphorylation by Src.^47^ In contrast, these results were not observed for the disease-associated mutants of PrP^C^. These findings suggest that PrP^C^ may have a role in both the phosphorylation of p190RhoGAP and RhoA-p190RhoGAP complex formation.

p190RhoGAP is activated by the binding of *β*1 integrins and then translocates into a detergent-insoluble fraction upon adhesion to fibronectin and colocalizes with F-actin in lamellipodial protrusions.^30, 48, 49^ Furthermore, integrin clustering triggers RhoA inactivation through c-Src-dependent activation of p190RhoGAP,^47^ and p190RhoGAP-mediated RhoA inactivation effectively induces neurite outgrowth in PC12 cells.^50^ In addition, PKA phosphorylates RhoA at Ser188, resulting in its release from membranes through increased interactions with RhoGDI.^51, 52^ Furthermore, the interactions between RhoA and RhoGDI were reported to negatively regulate the cycling of RhoA activity at the leading edge in migrating cells.^53^ We showed that overexpression of the RhoA S188D mutant but not the S188A mutant promoted neurite outgrowth in the NGF-treated PC12 cells expressing PrP^C^. These data indicate that PrP^C^ induced RhoA inactivation also through RhoA phosphorylation at Ser188. Furthermore, we demonstrated that PrP^C^ is colocalized with RhoA and that it enhanced the interaction between RhoA and p190RhoGAP in response to NGF. However, the interacting domains of PrP^C^ and RhoA remain to be elucidated. In general, active RhoA induced actin–myosin interactions, resulting in cell contraction, although inactive RhoA were reported to prevent actin–myosin interaction, which may induce cell expansion and neurite outgrowth.^54^

In prion diseases, genetic mutations of PrP^C^ induce spongiform encephalopathy and spontaneous neurodegeneration, and the disease-associated mutations of PrP^C^ lead to severe ataxia, apoptosis, and extensive central and peripheral myelin degeneration.^55, 56^ As shown in this study, overexpression of the disease-associated mutants of PrP^C^ (P102L and MΔ8) impaired neurite outgrowth because of the failure to inactivate RhoA and reduced the co-immunoprecipitation of RhoA and p190RhoGAP. Interestingly, scrapie infection increases RhoA activation by decreasing the interaction between RhoA and p190RhoGAP (manuscript in preparation). Based on these findings, the disease-associated mutations of PrP^C^ and scrapie infection partially suppress neuronal differentiation via the failure to inactivate RhoA.

Taken together, our results showed that PrP^C^ contributes to RhoA inactivation, leading to neuritogenesis and that disease-associated mutants of PrP^C^ failed to inactivate RhoA, which in turn leads to prion-related neurodegeneration. These findings are important for understanding the mechanisms of PrP^C^-mediated neuronal differentiation and survival.

## Materials and Methods

### Materials

Bovine serum albumin (BSA), Y27632, and the anti-*β*-actin antibody were purchased from Sigma-Aldrich (St. Louis, MO, USA). Anti-RhoA, anti-Rac1, anti-Cdc42, anti-RhoGDI, and anti-cofilin antibodies were obtained from Santa Cruz Biotechnology (Santa Cruz, CA, USA). NGF and the anti-p190RhoGAP antibody were purchased from Millipore (Lake Placid, NY, USA). Anti-p-RhoA (S188), anti-p-LIMK1/2, anti-LIMK1, and anti-LIMK2 antibodies were purchased from Abcam (Cambridge, MA, USA). The anti-p-cofilin antibody was obtained from Cell Signaling Technology (Danvers, MA, USA).

### Cell culture, transfection, and generation of stable cell lines

Mouse hippocampal neuronal cell lines, including ZW13-2 (WT PrP) and Zpl3-4 (PrP knockout) cells, were previously established.^26^ ZW and Zpl cells were maintained in Dulbecco's modified Eagle's medium (DMEM) (Hyclone, Logan, UT, USA) supplemented with 10% heat-inactivated fetal bovine serum (FBS; Hyclone), 100 units/ml penicillin and 100 *μ*g/ml streptomycin (Thermo Fisher Scientific, Rockford, IL, USA) at 37 °C under 5% CO_2_. Transient transfections were carried out using the Lipofectamine 2000 reagent (Thermo Fisher Scientific) according to the manufacturer's directions. For siRNA transfection, ZW cells were transfected with siRNA targeting human PrP (150 pmol/ml) for 72 h to silence PrP expression. PC12 cells stably expressing the pcDNA3.1/Zeo(+) vector or vector encoding human PrPs (WT; P102L, the most common GSS-causing mutation; MΔ8, octapeptide repeat deletions) were generated using the Lipofectamine 2000 reagent, followed by selection and maintenance in the presence of 250 *μ*g/ml Zeocin (Thermo Fisher Scientific). PC12 cells were grown in RPMI 1640 medium (Hyclone) supplemented with 10% heat-inactivated horse serum (HS, Hyclone), 5% FBS, 100 units/ml penicillin and 100 *μ*g/ml streptomycin at 37 °C under 5% CO_2_.

### Animals

The *Prnp*-transgenic (Tga20) and *Prnp*-deficient mice (Zürich I) were kindly provided by Dr. C Weissmann (Department of Infectology, Scripps Florida, Jupiter, FL, USA) and Dr. A Aguzzi (Institute of Neuropathology, University Hospital of Zürich, Zürich, Switzerland), respectively. The WT control male C57BL/6J mice were purchased from Young Bio (Seongnam, Republic of Korea). The Tga20, Zürich I and WT control C57BL/6J mice were housed in a clean facility under natural light-dark cycle conditions (12-h/12-h light/dark cycle) and examined at 8–10 weeks of age. All experiments were performed in accordance with Korean laws and with the approval of the Hallym Medical Center Institutional Animal Care and Use Committee (HMC2015-0-0411-3).

### Induction of neurite outgrowth in PC12 cells

To assess neurite outgrowth, the PC12 cells were plated at a density of 5 × 10^3^ cells per well on 35 mm culture dishes coated with poly-d-lysine solution (Sigma-Aldrich). After 12 h, the PC12 cells were incubated with 50 ng/ml NGF2.5S (Millipore) for the indicated times in DMEM medium containing with 1% heat-inactivated HS, 0.5% heat-inactivated FBS, and 100 units/ml penicillin and 100 *μ*g/ml streptomycin. The quantity of neurite bearing cells was determined by counting at least 100 single cells/3 arbitrary positions per dish. A cell was identified to as positive for neurite outgrowth if it had at least a twofold increased cell body diameter. Cells were visualized using a phase-contrast microscope (200x, Nikon TS100, Nikon, Tokyo, Japan).

### Western blot analysis

Cells were collected and washed once with ice-cold phosphate-buffered saline (PBS) and lysed with modified RIPA buffer (50 mM Tris-HCl (pH 7.4), 150 mM NaCl, 1% Nonidet P-40, 0.25% sodium deoxycholate, 10 mM NaF, 1 mM Na_3_VO_4_, 1 mM EDTA, and 1 mM EGTA) supplemented with a protease inhibitor cocktail tablet (Roche, Indianapolis, IN, USA). The cell lysates were centrifuged at 13 000 × *g* for 10 min, and the protein concentrations in the supernatants were analyzed using a BCA protein assay kit (Thermo Fisher Scientific). Equal amounts of proteins were separated using SDS-PAGE, transferred to PVDF membranes, and probed with the appropriate antibodies. Immunoreactive bands were visualized on digital images captured with an ImageQuant LAS4000 imager (GE Healthcare Life Sciences, Piscataway, NJ, USA) using EzwestLumi plus western blot detection reagent (ATTO Corporation, Tokyo, Japan), and the band intensities were quantified using ImageJ (NIH) program (Bethesda, MD, USA). Statistical analyses were performed using GraphPad Prism4 (San Diego, CA, USA).

### Immunocytochemistry

PC12 cells were treated with 50 ng/ml NGF2.5S in DMEM media (supplemented with 1% heat-inactivated HS, 0.5% heat-inactivated FBS, and antibiotics) for the indicated times at 37 °C under 5% CO_2_. The cells were washed with PBS and fixed with a 4% paraformaldehyde solution for 20 min at room temperature (RT). The cells were permeablized with 0.2% Triton X-100 for 10 min, and then the samples were blocked with 5% normal goat serum and 1% BSA in PBS for 15 min at RT. For fluorescence labeling, the cells were incubated with rabbit polyclonal anti-RhoA (1:100; Santa Cruz Biotechnology) and goat polyclonal anti-PrP (1:200; Santa Cruz Biotechnology) antibodies overnight at 4 °C. The cells were washed and incubated with fluorescein isothiocyanate-conjugated or rhodamine-conjugated anti-mouse or rabbit IgG (1:500) for 1 h, at RT. The immunolabeled cells were examined using a LSM 700 laser confocal microscope (Zeiss, Oberkochen, Germany).

### Immunoprecipitation

The cells were harvested and washed once with ice-cold PBS, and then lysed in modified RIPA buffer. The cell lysates were centrifuged for 10 min at 13 000 × *g* and the supernatants were incubated with anti-RhoA, anti-p190RhoGAP, and anti-PrP (3F10)^57^ antibodies for 2 h at 4 °C. After antibody binding, protein A-conjugated Sepharose 4B beads (Thermo Fisher Scientific) were added for 2 h at 4 °C. The beads were then washed three times with lysis buffer, and the bound proteins were eluted with 2 x Laemmli sample buffer by boiling. The samples were electrophoresed and analyzed by western blot with anti-RhoA, anti-p190RhoGAP, and anti-PrP (3F4 or 3F10)^57, 58^ antibodies.

### GST-Rhotekin-RBD pull-down assay for activating RhoA

The cells were harvested and washed with PBS, and then lysed in binding/washing/lysis buffer (25 mM Tris-HCl, pH 7.4, 150 mM NaCl, 5 mM MgCl_2_, 1% NP-40, 1 mM DTT, 5% glycerol, 10 mM NaF, 1 mM Na_3_VO_4_, 1 mM EDTA, and 1 mM EGTA) with a protease inhibitor cocktail tablet. The lysates were centrifuged at 13 000 × *g* for 10 min at 4 °C. The supernatant was incubated with GST-Rhotekin-RBD to detect RhoA-GTP. The beads were washed three times with binding/washing/lysis buffer. The bound proteins were eluted with 2 x Laemmli sample buffer by boiling. The samples were electrophoresed and analyzed by western blot with the anti-RhoA antibody.

### *In vitro* loading of GDP and GTP*γ*S onto GTP-binding proteins

Cell lysates (500 *μ*g/ml protein in 500 *μ*l) were incubated with 10 mM EDTA (pH 8.0). Next, 0.1 mM GTP*γ*S or 1 mM GDP was added to the cell lysates, and the lysates were incubated at 30 °C for 15 min under constant agitation. The reaction was terminated by thoroughly mixing the sample with MgCl_2_ at a final concentration of 60 mM on ice.

### *In vitro* GST-tagged protein–protein interactions

The purified recombinant GST and GST-RhoA proteins (10 *μ*g/ml protein in 500 *μ*l) were preincubated with glutathione (GSH)-sepharose 4B beads for 2 h at 4 °C in a binding buffer (50 mM Tris-HCl, pH 7.5, 1x PBS, and 10% glycerol,) with a protease inhibitor cocktail tablet. To determine protein–protein interaction, GST and GST-RhoA beads were incubated with 1–4 *μ*g of purified human recombinant PrP (Hu-PrP) for 2 h at 4 °C. After washing the beads, the bound proteins were eluted with 2 x Laemmli sample buffer by boiling. The samples were electrophoresed and analyzed by western blot with the anti-PrP antibody.

### Subcellular fractionation

Confluent cells were harvested, washed with ice-cold PBS, and lysed by passing through a 23-gauge syringe needle for 10 cycles in cold hypotonic buffer (10 mM Tris-HCl (pH 7.4), 1 mM DTT, 5 mM MgCl_2_, 10 mM KCl, 10 mM NaF, and 1 mM Na_3_VO_4_) with a protease inhibitor cocktail tablet. The lysates were centrifuged at 500 × *g* for 10 min. The pellets that contained nuclei and nuclei-associated structures were solubilized with HEPES buffer (pH 7.2) containing 400 mM NaCl, 1 mM EDTA, 1 mM DTT, and the protease inhibitor cocktail and were agitated on ice for 30 min. The postnuclear supernatants were centrifuged at 100 000 x *g* for 1 h at 4 °C to separate the membrane pellet and the cytosolic fraction. The membrane pellets were washed with ice-cold PBS and suspended in RIPA buffer by rocking for 1 h at 4 °C, followed by centrifugation at 13 000 x *g* for 10 min at 4 °C. The supernatant, containing the solubilized membrane proteins, was considered the membrane fraction.

### F-actin sedimentation assay

Cells were harvested and washed with PBS, and then lysed in 0.1% Triton X-100 and F-actin stabilization PHEM buffer (60 mM PIPES, 25 mM HEPES, 10 mM EGTA, 2 mM MgCl_2_, pH 6.9) with a protease inhibitor cocktail. The cell lysates were carefully mixed and directly transferred into a TLA 100 centrifuge tube (Beckman Instruments, Palo Alto, CA, USA). The lysates were centrifuged at 100 000 × *g* for 1 h at 4 °C in a table top ultracentrifuge (Beckman Instruments), which yielded a clear supernatant. At these high centrifugal forces, all F-actin in the system is expected to pellet, leaving G-actin in the supernatant. The F-actin pellet was washed twice in ice-cold PHEM buffer and suspended in SDS buffer. Protein concentration of the fractions was quantified using a BCA protein assay kit. Equal amounts of proteins were electrophoresed, and transferred to PVDF membrane for probing with anti-*β*-actin antibody. The densitometric quantification of the western blot determined the comparable levels of G- and F-actin using Image J software.

### Statistical analysis

The data are presented as the mean±S.E. of at least three independent experiments. Student's *t*-tests were used to compare groups using the GraphPad Prism4 program.
