# Supplementary figures and images for: Regulation of RhoA activity by the cellular prion protein
Source: Cell Death Dis. 2017 Mar 16;8(3):e2668–. doi: 10.1038/cddis.2017.37 (PMC5386549; doi:10.1038/cddis.2017.37)

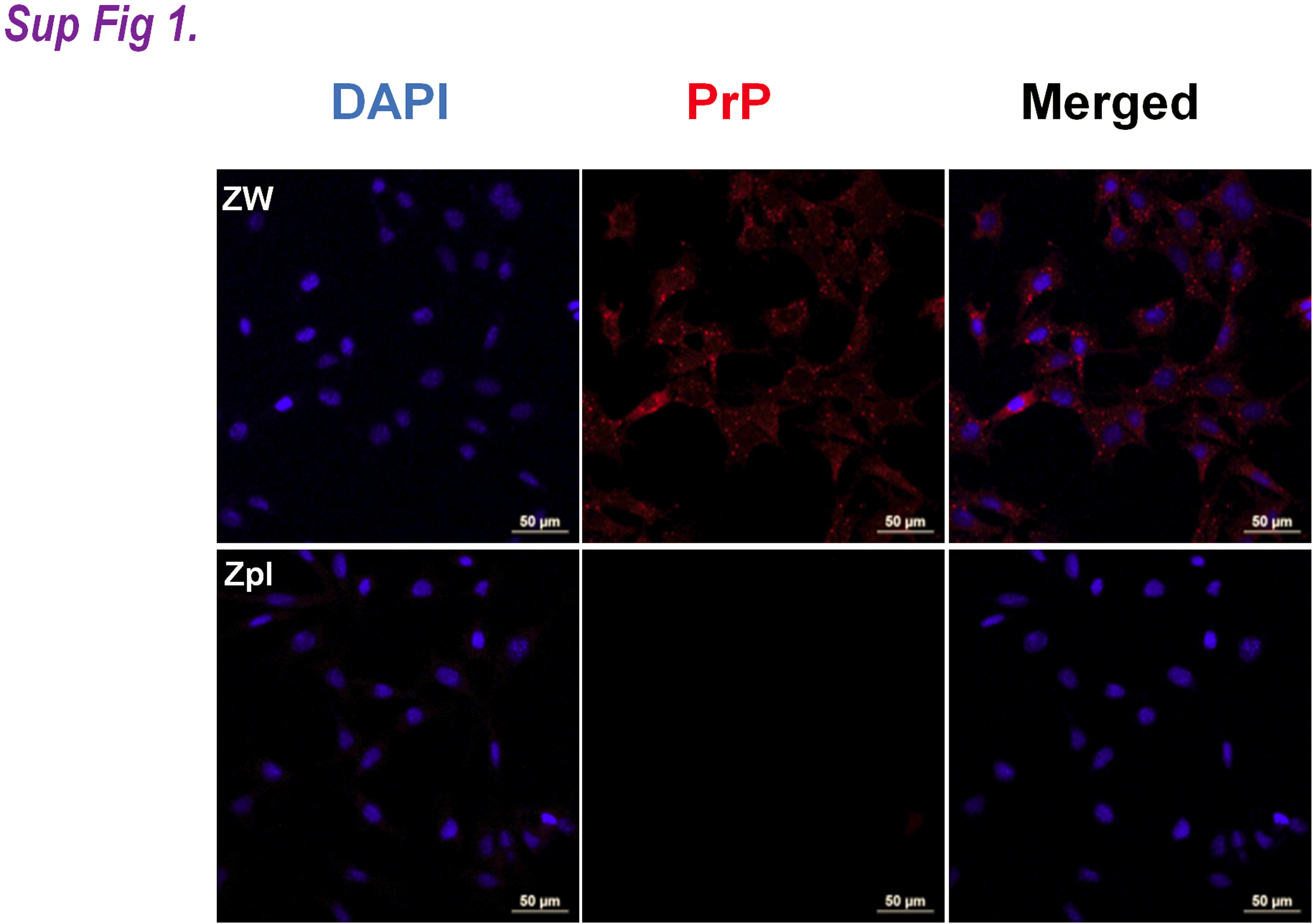

Supplement: Supplementary Figure 1 [file cddis201737x1.tif]

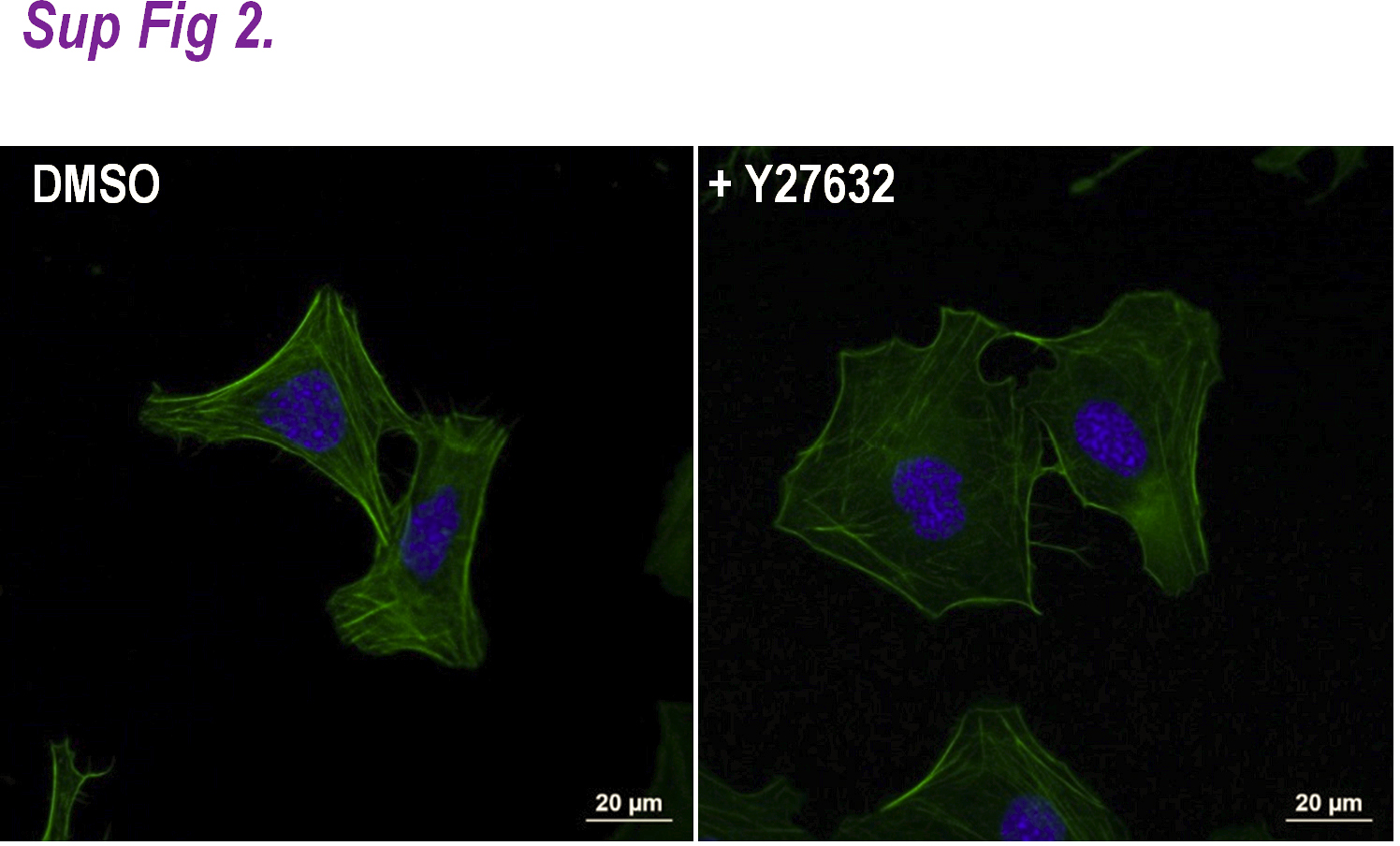

Supplement: Supplementary Figure 2 [file cddis201737x2.tif]

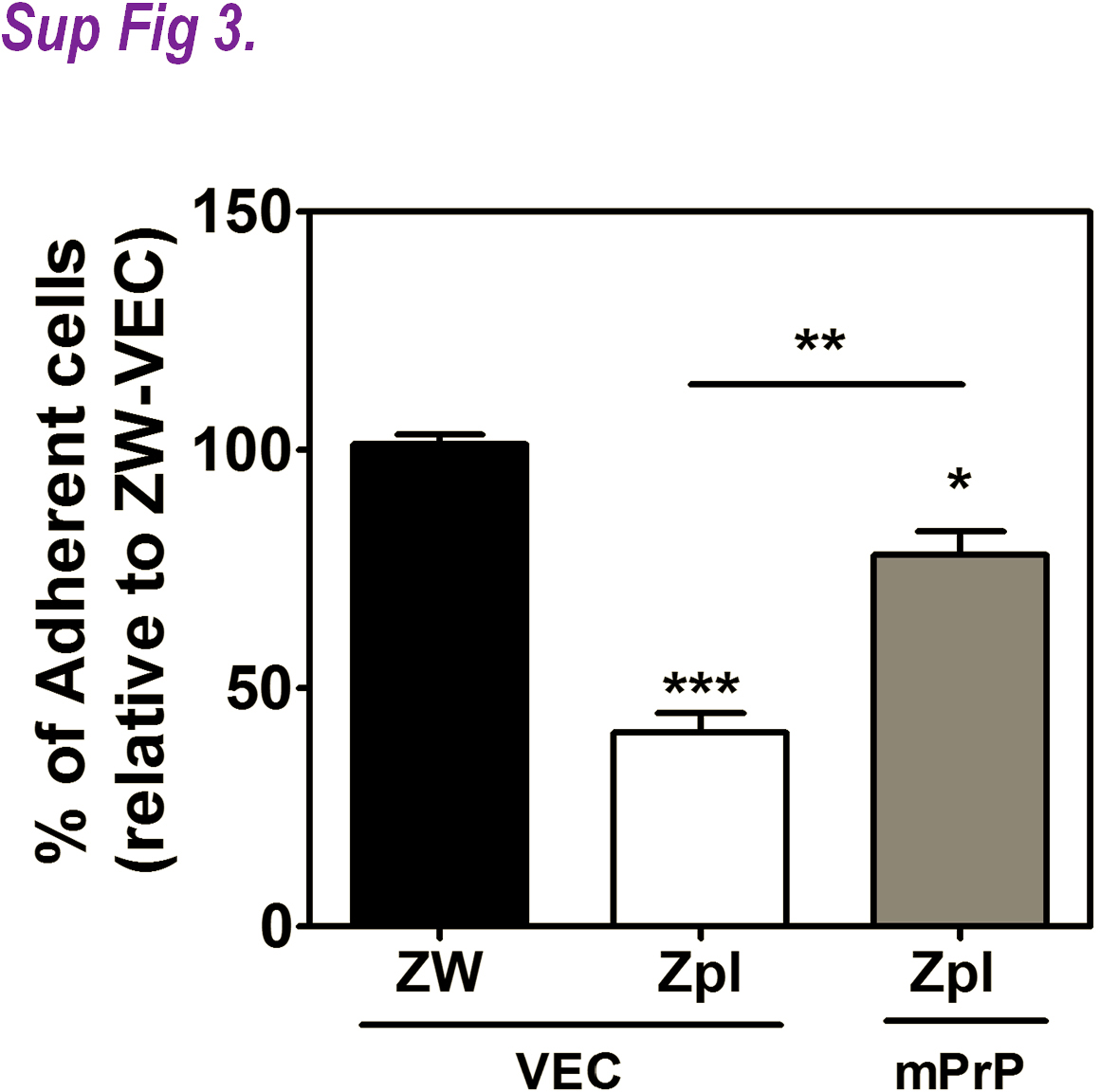

Supplement: Supplementary Figure 3 [file cddis201737x3.tif]

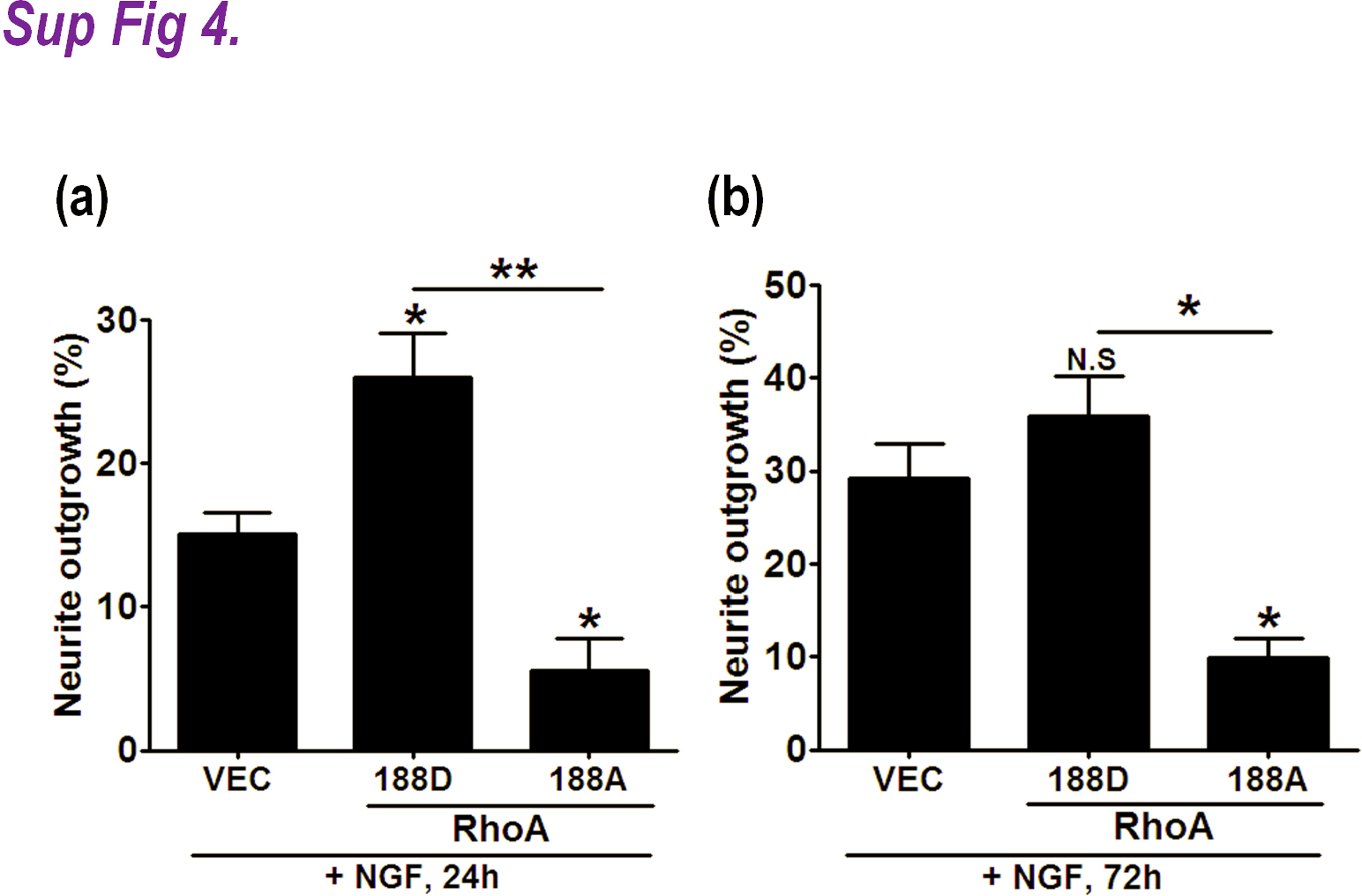

Supplement: Supplementary Figure 4 [file cddis201737x4.tif]
